# Genome-wide miR-155 and miR-802 target gene identification in the hippocampus of Ts65Dn Down syndrome mouse model by miRNA sponges

**DOI:** 10.1186/s12864-015-2160-6

**Published:** 2015-11-06

**Authors:** Xavier Bofill-De Ros, Mónica Santos, Maria Vila-Casadesús, Eneko Villanueva, Nuria Andreu, Mara Dierssen, Cristina Fillat

**Affiliations:** Institut d’Investigacions Biomèdiques August Pi i Sunyer (IDIBAPS), Rosselló 149-153, 08036 Barcelona, Spain; Centro de Investigación Biomédica en Red de Enfermedades Raras (CIBERER), Barcelona, Spain; Bioinformatics Platform, CIBERehd, Barcelona, Spain; Cellular and Systems Neurobiology, Systems Biology Programme, Centre for Genomic Regulation (CRG), Universitat Pompeu Fabra, Barcelona, Spain; Present address: Institute of Biology, Otto-von-Guericke University, Magdeburg, Germany

**Keywords:** miRNAs, Transcriptome, Sponges, Down syndrome

## Abstract

**Background:**

Down syndrome (DS) or trisomy 21 is the result of a genetic dosage imbalance that translates in a broad clinical spectrum. A major challenge in the study of DS is the identification of functional genetic elements with wide impact on phenotypic alterations. Recently, miRNAs have been recognized as major contributors to several disease conditions by acting as post-transcriptional regulators of a plethora of genes. Five chromosome 21 (HSA21) miRNAs have been found overexpressed in DS individuals and could function as key elements in the pathophysiology. Interestingly, in the trisomic Ts65Dn DS mouse model two of these miRNAs (miR-155 and miR-802) are also triplicated and overexpressed in brain.

**Results:**

In the current work, we interrogated the impact of miR-155 and miR-802 upregulation on the transcriptome of Ts65Dn brains. We developed a lentiviral miRNA-sponge strategy (Lv-miR155-802T) to identify *in vivo* relevant miR-155 and miR-802 target mRNAs. Hippocampal injections of lentiviral sponges in Ts65Dn mice normalized the expression of miR-155 and miR-802 and rescued the levels of their targets methyl-CpG-binding protein 2 gene (*Mecp2),* SH2 (Src homology 2)-containing inositol phosphatase-1 *(Ship1)* and Forkhead box protein M1 *(FoxM1)*. Transcriptomic data of Lv-miR155-802T miRNA-sponge treated hippocampi correlated with candidate targets highlighting miRNA dosage-sensitive genes. Significant associations were found in a subset of genes (*Rufy2*, *Nova1*, *Nav1*, *Thoc1* and *Sumo3*) that could be experimentally validated.

**Conclusions:**

The lentiviral miRNA-sponge strategy demonstrated the genome-wide regulatory effects of miR-155 and miR-802. Furthermore, the analysis combining predicted candidates and experimental transcriptomic data proved to retrieve genes with potential significance in DS-hippocampal phenotype bridging with DS other neurological-associated diseases such as Alzheimer’s disease.

**Electronic supplementary material:**

The online version of this article (doi:10.1186/s12864-015-2160-6) contains supplementary material, which is available to authorized users.

## Background

Down syndrome (DS) [OMIM 190685] is the result of an extra copy of chromosome 21 (HSA21). The phenotype of DS is characterized by a cognitive impairment and a plethora of symptoms affecting a wide range of organs [1]. It is likely that most of the DS phenotypes are related to alterations in gene expression. Changes in the transcriptome of DS have shown that about 29 % of the expressed chromosome 21 transcripts are overexpressed, the remaining being either compensated or highly variable between individuals [2]. This suggests the role of a selective group of genes to be central in DS phenotype. Supporting this view, it was recently proposed that one of the contributing mechanisms relies on the overexpression of few HSA21 genes modifying the chromatin environment of the nuclear compartment what leads to a general perturbation of the transcriptome [3]. However, little is known about the contribution of HSA21 overexpressed miRNAs to variations in the transcriptome.

Mouse models are valuable tools to study the impact of gene expression variation on the DS phenotype [4, 5]. In particular, the Ts65Dn is a model of DS that carries an extra chromosome spanning most of the region of MMU16 that is homologous to HSA21. The trisomic region extends from few kb upstream *Mrpl39* to *Zfp295* and contains roughly 136 genes and two miRNAs, miR-155 and miR-802 [6, 7]. Robust differences in the expression of thousands of genes in the cerebella of trisomic and euploid mice have been described to create a variable trisomic transcriptome [8]. Gene expression analysis of CA1 pyramidal neurons from the hippocampus of Ts65Dn and euploid mice identified alterations in transcripts relevant to neurodegeneration [9, 10]. MiRNA profiles in Ts65Dn mice identified 11 miRNAs differentially expressed between Ts65Dn and euploid suggesting their potential role in regulating a plethora of target genes [11]. In the current work, we propose a novel approach to study the contribution of overexpressed miRNAs to transcriptomic perturbations.

## Results and discussion

**Fig. 1:**
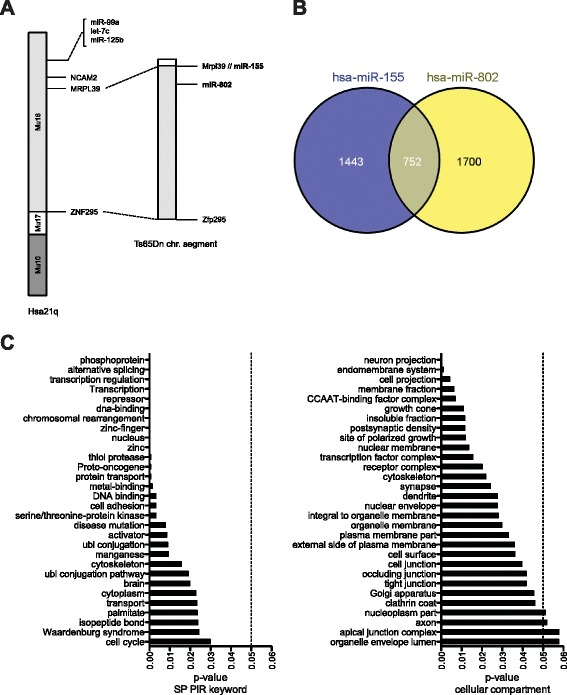
Predicted miR-155 and miR-802 targets are enriched in neuronal functions by gene ontology analysis. **a** Schematic representation of human chromosome 21 long-arm comprised of mouse chromosomal segments from chromosomes 16, 17, and 10. The portion of chromosome 16 that is found in trisomy in the Ts65Dn mouse expands from *Mrpl39* (mitochondrial ribosomal protein, human MRPL39) to *Zfp295* (zinc finger protein 295, human ZNF295) and harbors miR-155 and miR-802. **b** Venn diagram of hsa-miR-155 and hsa-miR-802 miRWalk predicted targets. **c** Functional profiling of the resulting list of 752 commonly predicted miR-155 and miR-802 targets in brain was analyzed using DAVID Bioinformatic Resources. Left panel shows enrichment in gene ontology terms related to SP PIR keywords. Right panel shows enrichment in gene ontology terms related to cellular compartment

To investigate the contribution of overexpressed miRNAs to the DS transcriptome, we have focused our study on the effects of miR-155 (MIRN155, miR155) and miR-802 (MIRN802, miR802), since both miRNAs are found in triple copy in the Ts65Dn mouse genome (Fig. 1a). A bioinformatic analysis of miR-155 and miR-802 predicted target genes, using miRWALK database, that combines information from 8 established miRNA predition programs [12], identified 2195 and 2452 potential targets, respectively, with an overlap of 752 genes (Fig. 1b). Gene ontology analysis of these putative commonly regulated transcripts using DAVID software (Database for Annotation, Visualization and Integrated Discovery [13]) ( Additional file 1: Table S1) identified an enrichment in genes involved in relevant GO terms such as phosphoproteins, neuron projections, synapses and dendrites, being all them key elements in neuronal physiology (Fig. 1c). This is interesting as Ts65Dn mice exhibit behavioral and cognitive dysfunction with alterations in different processes involved in memory and learning [14–16], features similar to those present in DS individuals.

To study the impact of miR-155 and miR-802 overexpression we have developed a strategy that consists in the use of miRNA sponge technology to sequester miR-155 and miR-802 and thereby prevent their binding to endogenous targets, resulting in normalization of gene expression of target genes in trisomic cells. The miRNA sponge engineered in viral vectors offer several advantages over the use of miRNA inhibitors oligonucleotides, such as good cellular delivery, sustained effects with no requirement of repeated injections and the potential of tissue specificity.

**Fig. 2:**
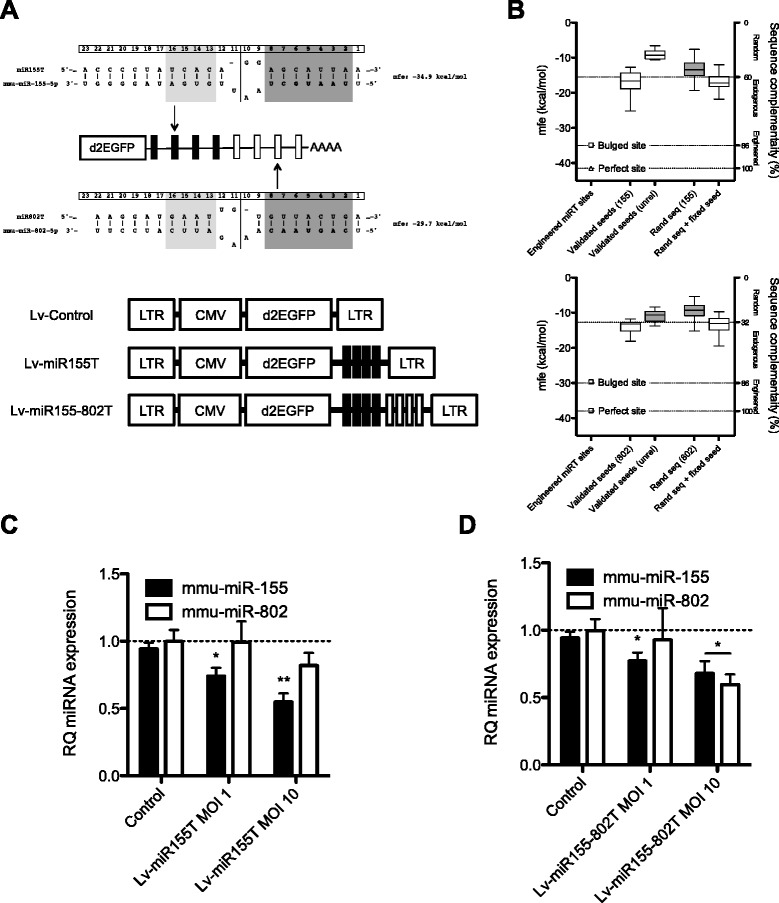
miR-155 and miR-802 expression are specifically reduced in cells infected with miRNA-sponge lentiviruses. **a** Top panel shows the sequence alignment of mmu-miR-155 and mmu-miR-802 to the corresponding miRNA sponge sites engineered in the lentiviral vectors. Bottom panel shows Lv-miRT constructs containing the CMV promoter to drive destabilized green fluorescent protein expression (d2EGFP) bearing 4 or 8 miRNA sponge sites in the 3′UTR. **b** Minimum free energy and complementarity displayed in the miRNA:target sites hybridizations: Engineered miRT sites (recognition of engineered miRNA target sites with perfect pairing or 10–11 mismatch), validated seeds (recognition of 18 miR-155 and 12 miR-802 sites from experimentally validated target genes by miR-155, and miR-802 or an unrelated miRNA), rand seq (recognition of 100 completely random sequences of 22 nucleotides by miR-155 and miR-802), rand seq + fixed seed (recognition of 100 random sequences of 22 nucleotides with fixed miR-155 and miR-802 seed region by miR-155 and miR-802). Energies were calculated using RNA hybrid algorithm. **c** HeLa cells stably expressing both mmu-miR-155 and mmu-miR-802 were transduced with 1 and 10 MOI of Lv-miR155T or Lv-Control. Expression of both miRNAs was analyzed 72-h later. RQ values represent mean ± SEM of four independent samples; *p < 0.05 and **p < 0.01 (Kruskal-Wallis test). **d** HeLa cells stably expressing both mmu-miR-155 and mmu-miR-802 were transduced with 10 MOI of Lv-miR155-802T or Lv-Control. Expression of both miRNAs was analyzed 72-h later. RQ values represent mean ± SEM of four independent experiments; *p < 0.05 (Kruskal-Wallis test)

We generated lentiviral vectors expressing EGFP with a 3′UTR containing 4 incomplete complementary binding sites recognizing miR-155 (Lv-miR155T) or both miR-155 and miR-802 (Lv-miR155-802T) (Fig. 2a). The sponge constructs were generated to contain bulged sites that are mispaired at positions 9–12, since it has been reported that such designs cannot mediate Ago2 cleavage of the transcript, allowing the miRNA binding to the transcript for longer, thus reducing its availability to regulate other transcripts [17, 18]. On the other hand this binding can stimulate the target-directed destabilization of the miRNA by the tailor and trimming processes leading to miRNA decay [19]. Despite the presence of mispaired nucleotides, the recognition of the designed target sites for the miRNAs was highly efficient since the minimum free energy calculated for the bulged sites was within −30 and −35 kcal/mol [20, 21] (Fig. 2b). The affinity for this incomplete binding showed to be far above from target sites of validated endogenous genes and close to the perfect pairing. To validate the capacity and specificity of the designed lentiviral vectors to modulate miR-155 and miR-802, HeLa cells generated to overexpress miR-155 and miR-802 (Additional file 2: Figure S1A, S1B) were transduced with different MOI of Lv-miR155T. MiRNA content was analyzed by RT-qPCR TaqMan assays and a dose-dependent reduction in miR-155 levels was observed whereas no changes were detected in miR-802 indicating the specificity of the effect (Fig. 2c). Moreover, HeLa cells overexpressing miR-155 and miR-802 when transduced with Lv-miR155-802T resulted in reduced expression of both miR-155 and miR-802 (Fig. 2d). Furthermore, the number of GFP expressing cells was substantially reduced in HeLa cells expressing miR-155 and miR-802 with respect to HeLa control cells (negative for miR-155 and miR-802) (Additional file 2: Figure S1C) when transduced with Lv-miR155T or Lv-miR155-802T. These results demonstrate that Lv-miR155-802T is a good tool to modulate miR-155 and miR-802 expression.

**Fig. 3:**
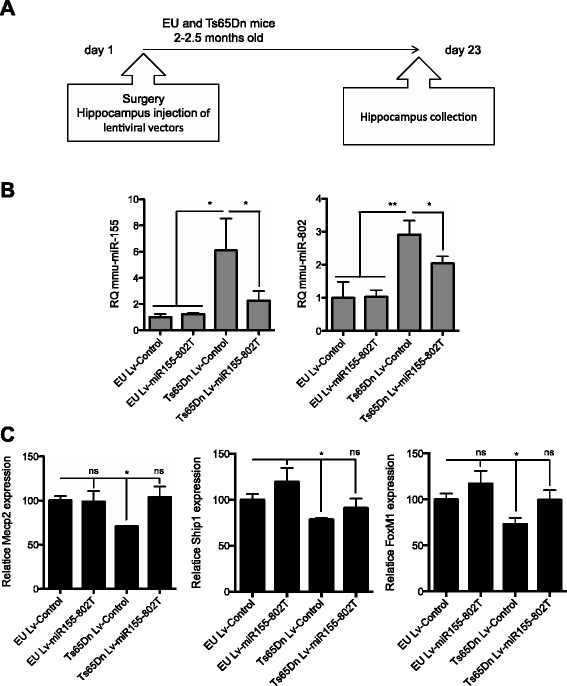
Decoy activity of Lv-miR155-802T significantly reduces hippocampal miR-155 and miR-802 content in Ts65Dn mice. **a** Schematic representation of the experimental design for local lentiviral infusion and sample collection. **b** Expression analysis by RT-qPCR (Applied Biosystems) of mmu-miR-155 (left panel) and mmu-miR-802 (right panel) in RNA extracts from hippocampus of euploid (EU) and Ts65Dn mice treated with Lv-Control or Lv-miR155-802T. RQ values represent mean ± SEM of six independent samples; *p < 0.05 and **p < 0.01 (Kruskal-Wallis test). **c** Expression analysis by RT-qPCR (Roche) of mmu-miR-155 and mmu-miR-802 validated target gene *Mecp2*, the mmu-miR-155 target *Ship1* and the mmu-miR-802 validated target *FoxM1* in RNA extracts from hippocampus of euploid (EU) and trisomic (Ts65Dn) adult mice treated with Lv-Control or Lv-miR155-802T. Values represent mean ± SEM of six independent samples; ns, non significant and * *p* < 0.05 and ** *p* < 0.01 (Kruskal-Wallis test)

To validate miRNA sponges as a strategy to identify miRNA targets *in vivo*, we stereotaxically infused in the ventral hippocampus of young adult (2–2.5 months old) euploid and Ts65Dn mice Lv-miR155-802T and Lv-Control viral particles. Three weeks upon infusion animals were sacrificed and miR-155 and miR-802 expression was analyzed (Fig. 3a). Increased miR-155 and miR-802 levels were detected in the hippocampus of Ts65Dn mice injected with Lv-Control with respect to euploid animals confirming previous findings [11]. Interestingly, the administration of Lv-miR155-802T resulted in a reduction in miR-155 and miR-802 content in Ts65Dn to levels similar to those of euploid mice. No effect was detected in control animals injected with the lentiviral sponge (Fig. 3b). In addition, we analyzed the expression levels of *Mecp2*, a previously validated target gene for both miRNAs [11], *Ship1* a miR-155 target gene [11] and *FoxM1* a miR-802 target [22, 23]. Expression analysis of *Mecp2*, *Ship1* and *FoxM1* showed a reduction in the hippocampus of trisomic mice that were rescued to wild type levels in Lv-miR155-802T injected animals (Fig. 3c).

**Fig. 4:**
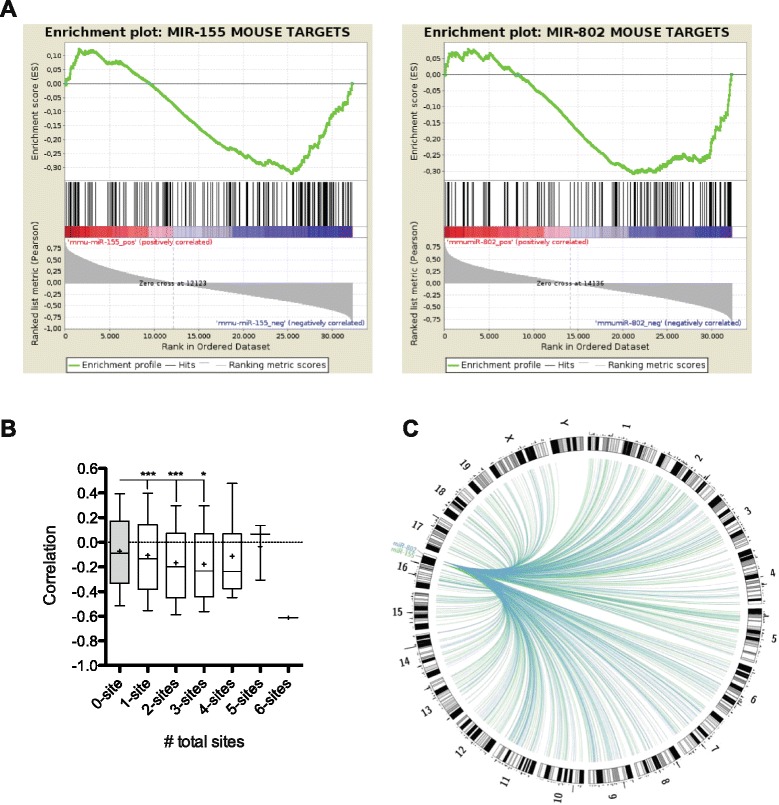
Transcriptome analysis reveals an enriched downregulation of miR-155 and miR-802 predicted targets. **a** Gene Set Enrichment Analysis of array data was performed on the set of mouse predicted targets for miR-155 (left panel) and miR-802 (right panel) in TargetScan6.2. **b** Analysis of gene expression explained by the number of predicted miRNA sites. Box plots represent the distribution of miRComb Pearson correlation coefficients among the targets sorted according to the predicted number of target sites (+ represent the mean of the distribution). * and *** denote *p* < 0.05 and *p* < 0.001, respectively. **c** Representation of the correlations between predicted target genes and miR-155 (green) and miR-802 (blue) according to its topology in mouse genome. The layer over the chromosomes represents in graphic bars the regulation of genes within subchromosomic segments of 1000 kb. Representation was performed using CIRCOS visualisation package. Represented correlations are those that presented negative coefficients for a predicted target and a *p*-value < 0.05 in miRComb analysis

To assess the transcriptomic impact of miR-155 and miR-802 overexpression we performed gene-expression profiling of euploid and Ts65Dn hippocampi treated with Lv-control or Lv-miR155-802T in an Agilent microarray platform [GEO: GSE68074]. Cluster analysis by principle component analysis (PCA) discriminated between genotypes but not between treatments (Additional file 2: Figure S2A), suggesting that the dosage imbalance effects on the steady state levels of thousands of genes was superior than the modulation by the Lv-miR155-802T sponge within each genotype. To assess the effects of Lv-miR-155-802T sponge we performed a gene set enrichment analysis [24] (GSEA) of array data on the set of miR-155 and miR-802 predicted target genes from TargetScan. We found enrichment in genes that negatively correlate with miR-155 or miR-802, consistent with miRNA activity (Fig. 4a). Further analysis on this enrichment was performed using miRComb R package, which calculated the correlation coefficients between signal intensity of transcripts in the microarray and miR-155 or miR-802 expression levels. This regression analysis considered all the treatment groups with variable levels of miR-155 or miR-802, involving genotype and treatment-based differences. The distributions of correlation scores showed that those genes with increased number of target sites display stronger negative correlation (Fig. 4b). This analytical approach combines sequence conservation parameters and experimental expression data, what refines miRNA target predictions integrating data from biological samples (Additional file 2: Figure S2B). Moreover, the topological analysis [25] of predicted genes with a significant negative correlation showed a genome-wide impact of miR-155 and miR-802 imbalance (Fig. 4c). Interestingly, when we constructed a topological map, based on the GEDDs defined by Letourneau and collaborators [3] plotted with our expression data, we could identify regions of coincidence from GEDDs to the expression associated to miR-155 and miR-802 deregulation (Additional file 2: Figure S3A). A 47 % of pairwise changes were estimated between the two studies. These data highlight that some of the epigenetic events can also be the consequence of the deregulation of epigenetic modulators such as MeCP2, a direct target of miR-155 and miR-802 [3]. These results point that the *in vivo* approach of lentiviral miRNA sponge is a good strategy to highlight the link of miR-155 and miR-802 levels regulating mRNA predicted targets. Of notice, lentivirus integration into the genome allows the analysis of steady-state transcriptomic variation derived from miRNA modulation. Interestingly, we performed a compared *in silico* analysis between our results and microarray data from GEO GSE47014 [26], in which the authors achieved a chromosome silencing of the extra HSA21 copy in trisomy 21 cells. In such experiments, presumably miR-155 and miR-802 expression should be normalized and impact to the global transcriptome. The results of this comparative analysis showed that 61–53 % genes predicted to be miR-155 and miR-802 targets based on our negative correlations showed downregulation in trisomy 21 cells, and more interestingly from the same miRNA prediction lists 59–58 % presented a normalization of its expression upon extra HSA21 silencing (Additional file 2: Figure S3B). Interestingly, the TargetScan Platform facilitated further analyses to show the number and the conservation of miRNA target sites within the transcript and to infer regulatory networks between mouse and human cells.

**Fig. 5:**
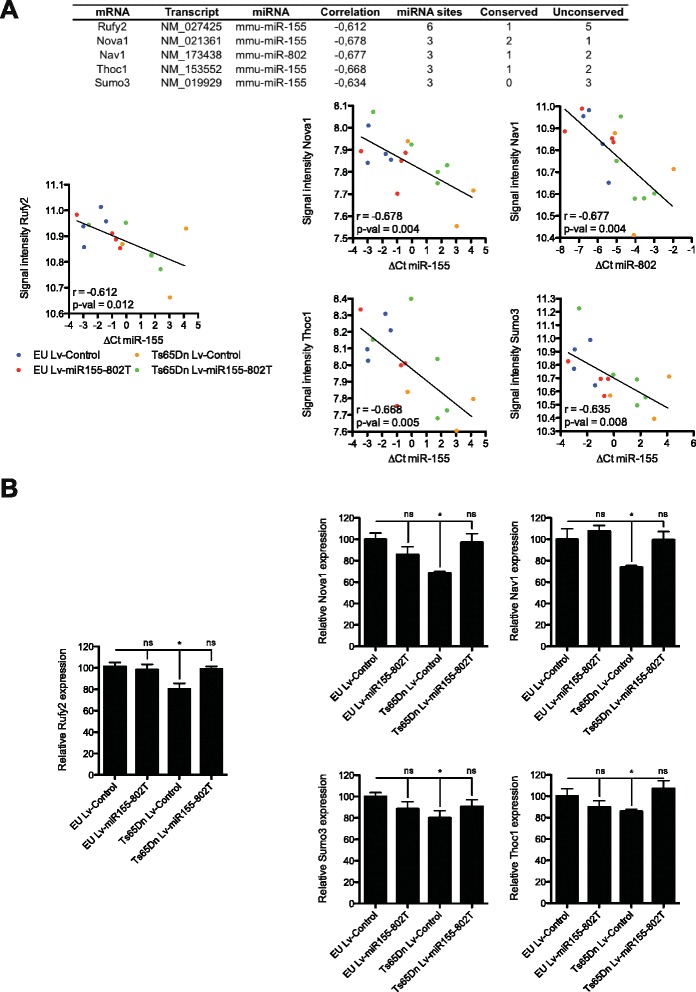
Validation of novel target genes contributing to T565Dn phenotype by RT-PCR. **a** Upper panel shows a table with five predicted target genes selected based on the criteria of predicted number of target sites and strong correlation coefficient (*Rufy2*, *Nova1*, *Nav1*, *Thoc1*, *Sumo3*). Table shows correlation values and the number of predicted miRNA target sites classified according its evolutionary conservancy. Below panel shows the correlation plots calculated using miRComb package for the previously selected target genes. **b** Expression analysis by RT-qPCR (Roche) of miR-155 and miR-802 target genes in RNA extracts from hippocampus of euploid (EU) and trisomic (Ts65Dn) adult mice treated with Lv-Control or Lv-miR155-802T. Data normalized to the expression of GdX (also named Ubl4A) housekeeping gene. Values represent mean ± SEM of six independent samples. * and ns denote *p* < 0.05 and not significant, respectively (Kruskal-Wallis test)

Further analyses of transcriptomic changes associated to miR-155 and miR-802 expression were conducted to identify those predicted mRNAs more likely to be directly regulated. To this end gene sets were sorted according to the number of predicted miRNA target sites in the 3′UTR and the correlation score (Additional file 3: Table S2). A subset of five genes, on the top of that list, were selected for validation (Fig. 5a). Quantitative RT-qPCR data confirmed downregulation of *Rufy2*, *Nova1*, *Nav1*, *Thoc1* and *Sumo3* in the hippocampi of Ts65Dn mice and their restored expression in Ts65Dn Lv-miR-155-802T treated mice (Fig. 5b). Interestingly, all five genes are involved in neuronal function and cellular homeostasis, with relevant implications to neurological diseases. For instance, RUFY2 (*RUN and FYVE domain-containing 2*) belongs to RUFY family, which is involved in the regulation of neuronal polarity and membrane trafficking [27]. Moreover, RUFY2 has been implicated in the reduction of beta-amyloid secretion in late-onset Alzheimer’s disease [28]. Similarly, SUMO3 (Small ubiquitin-like modifier 3) has also been linked to Alzheimer’s disease, since endogenous protein sumoylation activity reduces beta-amyloid production [29]. NOVA1 (*Neuro-oncological Ventral Antigen 1*) is a neuron-specific splicing factor that controls the alternative processing of a large set of mRNAs important for the synaptic activity [30]. The defect in neuronal viability observed in Nova-1 null mice suggests either a loss of function or a gain of function derived splicing defects in inhibitory receptors GABA(A)Rgamma2, GlyRalpha2, that could trigger imbalances between inhibition and excitation signals [31]. In this line, treatment of Ts65Dn mice with GABA(A) antagonists have shown a rescue of cognitive defects and improved long-term potentiation [32]. NAV1 (*Neuron navigator 1*) is a microtubule-associated protein involved in neuronal migration [33] and reorganization of the cytoskeleton to induce neurite-like extensions [34]. Accordingly, reduced neurite length of neuronal precursors was observed in Ts65Dn mice [35]. Finally, THOC1 (*THO Ribonucleoprotein Complex 1*) is a ribonucleoprotein involved in mRNA elongation essential for early embryonic development [36], however its contribution to neuronal homeostasis is still unclear. Whether the transcriptomic rescue of the perturbed gene set in the hippocampi of Ts65Dn mice upon lentiviral Lv-miR-155-802T infusion may have any impact in brain physiology or the cognitive deficits of Ts65Dn mice remains to be studied. However, it could be speculated that normalization of the expression levels of the key target genes identified will be of functional relevance. In fact, normalization of DYRK1A or SNX27 expression in the hippocampi of adult Ts65Dn mice by strategies based on viral vector administration has shown rescue of synaptic deficits and partial recovery of behavioural abnormalities [37, 38].

The potential therapeutic effect of the sponge approach could also be of interest to be explored in other diseases by which functional miRNA-disease associations have been identified such as cancer, Type II diabetes, Asthma or Alzheimer disease [39].

## Conclusions

The present study establishes a link between overexpression of miR-155 and miR-802 and downregulation of a new set of candidate genes with potential contribution to DS neuronal function. The miRNA sponge developed strategy provides a useful tool for the identification of subtle transcriptomic changes, often masked for interindividual variability. These results also highlight that DS hippocampal-dependent phenotypes have connections with alterations present in neurological-associated diseases such as Alzheimer and suggests that modulation of miRNAs could be envisioned as a novel pharmacotherapy approach in DS.

## Methods

### Cells lines and cell culture

HeLa and HEK293T cells were obtained from the American Type Culture Collection (ATCC). Cells were maintained in Dulbecco’s modified Eagle’s medium (Gibco BRL), supplemented with 10 % fetal bovine serum, penicillin (100 mg/ml) and streptomycin (100 mg/ml) (Gibco BRL).

HeLa cells stably expressing mmu-miR-155 and mmu-miR-802 were generated by calcium phosphate transfection (Clontech) of p-miR155 and p-miR802 and selection with 0.2 mg/ml hygromycin and 0.4 mg/ml G418 respectively.

### Constructs

Oligonucleotide sequences were designed with four imperfect target sites. Three nucleotides mismatch were introduced between positions 10 and 11 from the miRNA seed sequences to prevent AGO2-mediated mRNA cleavage and therefore trigger miRNA decoy effects. miR155T (sense 5′ CGATACCCCTATCACAGGAGCATTAAATGCACCCCTATCACAGGAGCATTAAGTACACCCCTATCACAGGAGCATTAATGCAACCCCTATCACAGGAGCATTAAT 3′) contained restriction sites for ClaI and XbaI, and miR802T (sense 5′ AATTCAAGGATGAATTGTGTTACTGAATGCAAGGATGAATTGTGTTACTGAGTACAAGGATGAATTGTGTTACTGATGCAAAGGATGAATTGTGTTACTGAGGTAC 3′) contained restriction sites for EcoRI and KpnI. These oligonucleotides were chemically synthesized, HPLC purified and purchased (Bonsaitech). Sense and antisense oligonucleotides were annealed and cloned in the pSP72 expression vector (Promega). miR155T and miR802T were further cloned in the pd2EGFP-C1 vector containing a destabilized form of the EGFP protein with two PEST domains (kindly provided by Dr. Raúl Méndez). Finally the d2EGFP-miRT sequences were cloned in the pLS-CG vector (Addgene #12161) at the AgeI KpnI sites to generate pLv-miR155T and pLv-miR155-802T.

mmu-miR-155 and mmu-miR-802 (p-miR155 and p-miR802) expression vectors were generated by PCR of the genomic region containing the mature microRNA sequence (400 bp and 447 bp respectively) and cloning into the pLHCX and pLXSN retroviral vectors (Clontech), respectively.

### Lentivirus generation and titration

Lentiviruses were generated in HEK293T cells by calcium phosphate transfection (Clontech) of the following plasmids: pCMVAR8.91, pVSV-G and pLv-Control (empty vector) or pLv-miR155T or pLv-miR155-802 T. Resulting supernatants of HEK 293T transfected cells were collected at 72 h and concentrated by ultracentrifugation (2 h at 12 °C at 20200 r.p.m). After, the pellet was resuspended in PBS for 16 h at 4 °C and stored at −80 °C.

Viral titration was performed by qPCR with specific primers (Fw 5′- ACCTGAAAGCGAAAGGGAAAC-3′, and Rev 5′-CACCCATCTCCTCCTTCTAGCC-3′). Resulting Cts were interpolated in a standard curve that was generated by serial dilutions of pLS-CG mixed in a background of genomic DNA. The number of molecules was calculated using the formula: M = (C x 6, 02 x 10^23^)/(660 x bp), where C is the concentration of the plasmid, bp is the number of base pairs and M is the number of lentiviral transducing units.

### Quantitative miRNA RT-PCR

Total RNA was obtained from cell cultures or tissues using miRNeasy Mini RNA Extraction Kit (Qiagen). A total of 10 ng total RNA were reverse transcribed using a reverse transcriptase and stem-loop primers as indicated by the manufacturer (TaqMan MicroRNA Reverse Transcription Kit, Applied Biosystems). One and a half microliters of the reaction was used as a template for the qPCR amplification reaction (TaqMan Universal Master Mix, No AmpErase UNG, Applied Biosystems) in a thermocycler (ViiA 7 Real-Time PCR system, Applied Biosystems). Quantitative miRNA RT-PCR expression data were normalized to small nucleolar RNA U6 expression (RNU6B). Stem-loop primers and qPCR probes were purchased from TaqMan MicroRNA assay (Applied Biosystems): RNU6B (AB ID: 001093), mmu-miR-155 (AB ID: 002571), mmu-miR-802 (AB ID: 002029).

### Stereotaxic injection

Euploid and Ts65Dn young adult mice (2 to 2.5 month-old) were anesthetized with a combination of 1 mg/Kg medetomidine (Domtor, Pfizer) and 75 mg/Kg ketamine (Imalgene 500, Merial), and immobilized in a stereotaxic frame (Harvard Apparatus). Bilateral injections were performed at the level of the ventral hippocampus at the following coordinates relative to bregma (anterior-posterior = −3.3 mm, medial-lateral = +/− 3 mm, dorso-ventral = −3.3 mm and −2.3 mm) using a 5 μl Hamilton syringe. Up to 1008 transducing units (3 μl of viral suspensions of Lv-Control or Lv-miR155-802T) were injected into each hemisphere at a rate of 0.2 μl/min, under the precise control of an infusion pump (Ultramicropump, World Precision Instruments). The needle was left in place for 5 min after injection and then slowly retracted from the brain. Before complete withdrawal, the needle was allowed to dwell for an additional 5 min. After surgical intervention, the animals were injected subcutaneously with a dose of 2 mg/kg of atipamezole (Antisedan, Pfizer) for anaesthetic reversal. The analgesic, buprenorphine (Buprex, ScheringPlough) was also administered intraperitoneally at a dose of 0.05 mg/Kg twice a day for the following 72 h after intervention. Mice were euthanized and the hippocampus dissected at day 23 after infusion.

### Microarray expression analysis

Transcriptome of hippocampus of euploid and trisomic mice treated with Lv-Control or Lv-miR155-802T (*n* = 3–5 in each group) was analysed using an *Agilent* SurePrint G3 Mouse gene expression 8 x 60 K Microarray (ID 028005). A total RNA 100 ng were labeled using LowInputQuick Amp Labeling kit (Agilent 5190–2305) following manufacturer instructions. Briefly: mRNA was reverse transcribed in the presence of T7-oligo-dT primer to produce cDNA. cDNA was then *in vitro* transcribed with T7 RNA polymerase in the presence of Cy3-CTP to produce labeled cRNA. The labeled cRNA was hybridized to the microarray according to the manufacturer’s protocol. The arrays were washed, and scanned on an Agilent G2565CA microarray scanner at 100 % PMT and 3 μm resolution. Intensity data was extracted using the Feature Extraction software (Agilent).

### miRComb analysis

Correlation coefficients between gene expression and miRNAs were calculated using miRComb R package (http://mircomb.sourceforge.net). Expression was obtained from array-based intensity signal followed by quantile normalization by limma [40] and batch correction through ComBat [41]. MiR-155 and miR-802 expression (ΔCt) was analysed as previously described by RT-qPCR. Pearson Correlation coefficients were computed for each miRNA-mRNA pair. Only miRNA-mRNA pairs that showed significant (*p* < 0.05) negative correlation and were predicted to have at least one target site (according to TargetScan6.2 [42]) were considered for further analysis.

### cDNA synthesis and real-time quantitative PCR

RNA was isolated using miRNeasy Mini Kit (Qiagen). A total of 1 μg was reverse transcribed using Moloney Murine Leukemia Virus reverse transcriptase and oligo(dT) (Ambion). One microliter of the reaction was used as a template for the qPCR amplification reaction (LightCycler 480SYBER Green I Master Mix, Roche) in a thermocycler (ViiA 7 Real-Time PCR system, Applied Biosystems). *Mecp2*: Fw 5′- GGGAAGCCTCTGAGACCCTA-3′, and Rev 5′-CCTACCTGTCA GTGGCCAAG-3′. *Ship1*: Fw 5′- CGGTTTCATCTTC CACAGCCAAC-3′, and Rev 5′- GCTTCCACCTTTCCCAGATCC-3′*. FoxM1*:Fw 5′- CACTTGGATTGAGGACCACTT-3′, and Rev 5′- GTCGTTTCTGCTGTGATTCC-3′. *Rufy2*: Fw 5′- TGGGCTTAAGACTCCGCTTG −3′, and Rev 5′-GAATGGAGGCTGCTGACTGT −3′. *Nova1*: Fw 5′-AGCAGAACGGGACCCATACG-3′, and Rev 5′-CCTGGATCAAGCAAACCCTCT-3′. *Nav1*: Fw 5′- TGCCAACGCTAACCTAGTGG-3′ and Rev 5′- GCTTTGTTGAAGGAACTCCGA-3′. *Thoc1*: Fw 5′-AACCAAGATGTCTCCGACGC-3′ and Rev 5′-ACAAATGGTGTGGACGCAGT-3′. *Sumo3*: Fw 5′-GCAACCATGTCGGAAGAGAAG-3′ and Rev 5′-TGGTTGTCCATCAAACCGGA-3′. Quantitative RT-PCR expression data were normalized to *Gdx*: Fw 5′-GGCAGCTGATCTCCAAAGTCCTGG-3′, and Rev 5′-AACGTTCGATGTCATCCAGTGTTA-3′.

## Additional files

Additional file 1: Table S1. Gene ontology analysis of predicted transcripts. (XLSX 104 kb) Additional file 2: Fig. S1. a) Schematic representation of lentiviral vectors used to overexpress mmu-miR-155 and mmu-miR-802 (p-miR155 and p-miR802). b) Expression of mmu-miR155 and miR-802 in HeLa cells transfected with p-miR155 and p-miR802 (HeLa-miR-155-miR802). c) d2EGFP expression in HeLa and HeLa-miR-155-miR802 transduced with Lv-Control Lv-miR155T or Lv-miR155-802T. Top panel shows representative images of d2EGFP positive cells at 72-hours after transduction with 10 MOI of Lv-Control, LvmiR155Tor Lv-miR155-802T. Bottom panel shows the number of d2EGFP positive cells at different viraldoses. Image analysis was assessed using ImageJ software. **Fig. S2.** a) Principal component analysis of data obtained with the Agilent SurePrint G3 Mouse gene expression 8x60K Microarray (ID 028005). TheGEO accession number for our microarray data reported in this paper is GSE68074. Table of meandistances among the different genotypes and treatments. b) Prediction of miRNA targets based oncorrelation coefficients obtained from hippocampus treatment with sponge lentiviruses by different methods.Left panel shows the correlation prediction compared to the number of target sites predicted by TargetScan.Central panel shows the correlation prediction compared to the probability of conserved targeting(TargetScan PCT score). Right panel shows the correlation prediction compared to TargetScan contextscore. **Fig. S3.** a) Representation of Down’s syndrome gene expression dysregulation domanins (GEDDs)described by Letourneau and collaborators (black barplot overlay), and changes in expression in the miceorthologous genes observed in hippocampus upon the treatment with Lv-miR155-802T (grey barplotoverlay). Representation was performed using CIRCOS visualization package. b) Venn diagram of miRNApredicted targets on Lv-miR155-802T treated hippocampus based on the negative coefficients obtainedfrom miRComb analysis package for miR-155 (Corr_155 -) and miR-802 (Corr_802 -), genes that presenteda negative fold-change in between euploid and trisomic condition (TS/WT -) and genes that presented apositive fold-change in tisomic cells upon the XIST-induced HSA21 silencing (TS-XIST/TS +). (DOCX 3411 kb) Additional file 3: Table S2. miRComb correlation scores. (XLSX 62 kb)

## Abbreviations

HSA21: Human chromosome 21
MMU16: Murine chromosome 16
Ts65Dn: Trisomic mice for a segment of mouse chromosome 16 and mouse chromosome 17 orthologous to HSA21
miRNA: microRNA
