# Supplementary material for: Genome-wide miR-155 and miR-802 target gene identification in the hippocampus of Ts65Dn Down syndrome mouse model by miRNA sponges
Source: BMC Genomics. 2015 Nov 6;16:907. doi: 10.1186/s12864-015-2160-6 (PMC4636806; doi:10.1186/s12864-015-2160-6)
Supplement: Additional file 2: Fig. S1. — a) Schematic representation of lentiviral vectors used to overexpress mmu-miR-155 and mmu-miR-802 (p-miR155 and p-miR802). b) Expression of mmu-miR155 and miR-802 in HeLa cells transfected with p-miR155 and p-miR802 (HeLa-miR-155-miR802). c) d2EGFP expression in HeLa and HeLa-miR-155-miR802 transduced with Lv-Control Lv-miR155T or Lv-miR155-802T. Top panel shows representative images of d2EGFP positive cells at 72-hours after transduction with 10 MOI of Lv-Control, LvmiR155Tor Lv-miR155-802T. Bottom panel shows the number of d2EGFP positive cells at different viraldoses. Image analysis was assessed using ImageJ software. Fig. S2. a) Principal component analysis of data obtained with the Agilent SurePrint G3 Mouse gene expression 8x60K Microarray (ID 028005). TheGEO accession number for our microarray data reported in this paper is GSE68074. Table of meandistances among the different genotypes and treatments. b) Prediction of miRNA targets based oncorrelation coefficients obtained from hippocampus treatment with sponge lentiviruses by different methods.Left panel shows the correlation prediction compared to the number of target sites predicted by TargetScan.Central panel shows the correlation prediction compared to the probability of conserved targeting(TargetScan PCT score). Right panel shows the correlation prediction compared to TargetScan contextscore. Fig. S3. a) Representation of Down’s syndrome gene expression dysregulation domanins (GEDDs)described by Letourneau and collaborators (black barplot overlay), and changes in expression in the miceorthologous genes observed in hippocampus upon the treatment with Lv-miR155-802T (grey barplotoverlay). Representation was performed using CIRCOS visualization package. b) Venn diagram of miRNApredicted targets on Lv-miR155-802T treated hippocampus based on the negative coefficients obtainedfrom miRComb analysis package for miR-155 (Corr_155 -) and miR-802 (Corr_802 -), genes that presenteda negative fold-change in between [file 12864_2015_2160_MOESM2_ESM.docx]

**Supplementary Figures**

**Fig. S1**

A) Schematic representation of lentiviral vectors used to overexpress mmu-miR-155 and mmu-miR-802 (p-miR155 and p-miR802).

(B) Expression of mmu-miR155 and miR-802 in HeLa cells transfected with p-miR155 and p-miR802 (HeLa-miR-155-miR802).

(C) d2EGFP expression in HeLa and HeLa-miR-155-miR802 transduced with Lv-Control Lv-miR155T or Lv-miR155-802T. Top panel shows representative images of d2EGFP positive cells at 72-hours after transduction with 10 MOI of Lv-Control, Lv-miR155T or Lv-miR155-802T. Bottom panel shows the number of d2EGFP positive cells at different viral doses. Image analysis was assessed using ImageJ software.

**Fig. S2**

(A) Principal component analysis of data obtained with the Agilent SurePrint G3 Mouse gene expression 8x60K Microarray (ID 028005). The GEO accession number for our microarray data reported in this paper is GSE68074. Table representing the mean distances between the individuals of the different treatments and genotypes with respect of each population mean.

(B) Prediction of miRNA targets based on correlation coefficients obtained from hippocampus treatment with sponge lentiviruses by different methods. Left panel shows the correlation prediction compared to the number of target sites predicted by TargetScan. Central panel shows the correlation prediction compared to the probability of conserved targeting (TargetScan PCT score[[1](#_ENREF_1)]). Right panel shows the correlation prediction compared to TargetScan context score[[2](#_ENREF_2)].

**
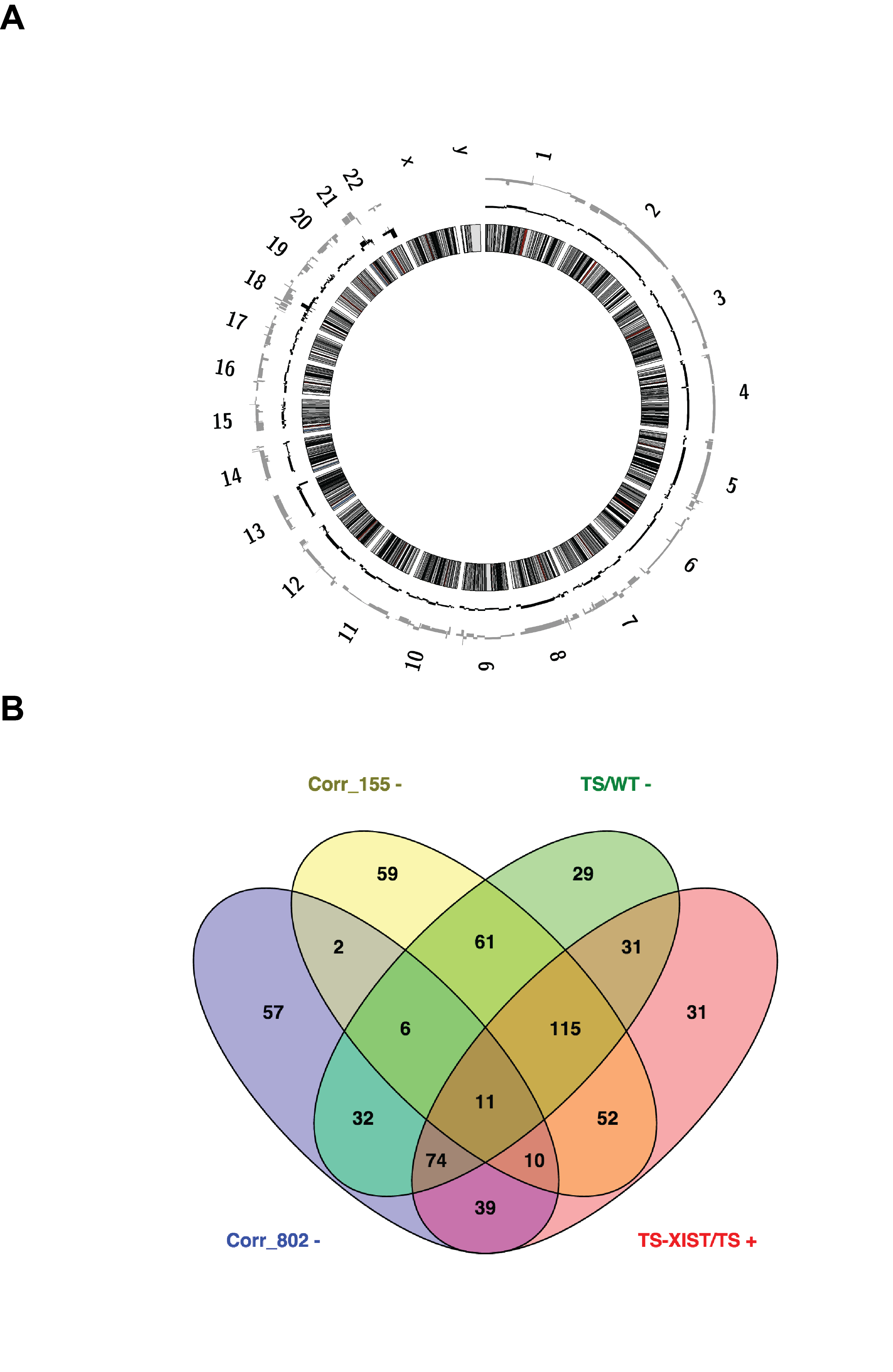
**

**Fig. S3**

(A) Representation of Down’s syndrome gene expression dysregulation domanins (GEDDs) described by Letourneau and collaborators[[3](#_ENREF_3)] (black barplot overlay), and changes in expression in the mice orthologous genes observed in hippocampus upon the treatment with Lv-miR155-802T (grey barplot overlay). Representation was performed using CIRCOS visualization package[[4](#_ENREF_4)].

(B) Venn diagram of miRNA predicted targets on Lv-miR155-802T treated hippocampus based on the negative coefficients obtained from miRComb analysis package for miR-155 (Corr_155 -) and miR-802 (Corr_802 -), genes that presented a negative fold-change in between euploid and trisomic condition (TS/WT -) and genes that presented a positive fold-change in tisomic cells upon the XIST-induced HSA21 silencing (TS-XIST/TS +)[[5](#_ENREF_5)].

**Table S1. Gene Ontology analysis of predicted transcripts**

**Table S2. miRComb correlation scores**

**Supplementary References**

1. Friedman RC, Farh KK, Burge CB, Bartel DP: **Most mammalian mRNAs are conserved targets of microRNAs**. *Genome Res* 2009, **19**(1):92-105.

2. Grimson A, Farh KK, Johnston WK, Garrett-Engele P, Lim LP, Bartel DP: **MicroRNA targeting specificity in mammals: determinants beyond seed pairing**. *Mol Cell* 2007, **27**(1):91-105.

3. Letourneau A, Santoni FA, Bonilla X, Sailani MR, Gonzalez D, Kind J, Chevalier C, Thurman R, Sandstrom RS, Hibaoui Y *et al*: **Domains of genome-wide gene expression dysregulation in Down's syndrome**. *Nature* 2014, **508**(7496):345-350.

4. Krzywinski M, Schein J, Birol I, Connors J, Gascoyne R, Horsman D, Jones SJ, Marra MA: **Circos: an information aesthetic for comparative genomics**. *Genome Res* 2009, **19**(9):1639-1645.

5. Jiang J, Jing Y, Cost GJ, Chiang JC, Kolpa HJ, Cotton AM, Carone DM, Carone BR, Shivak DA, Guschin DY *et al*: **Translating dosage compensation to trisomy 21**. *Nature* 2013, **500**(7462):296-300.
